# Lower serum magnesium is associated with vascular calcification in peritoneal dialysis patients: a cross sectional study

**DOI:** 10.1186/s12882-017-0549-y

**Published:** 2017-04-06

**Authors:** Amber O. Molnar, Mohan Biyani, Ian Hammond, John Paul Harmon, Susan Lavoie, Brendan McCormick, Manish M. Sood, Jessica Wagner, Elena Pena, Deborah L. Zimmerman

**Affiliations:** 1 Division of Nephrology, Department of Medicine, McMaster University, Hamilton, ON Canada; 2 Department of Radiology, University of Ottawa, Ottawa, ON Canada; 3 Department of Medical Imaging, The Ottawa Hospital Ottawa, Ottawa, ON Canada; 4 Division of Nephrology, Department of Medicine, Northern Ontario School of Medicine, Sudbury, ON Canada; 5 Kidney Research Centre, Ottawa Hospital Research Institute, Ottawa, ON Canada; 6 The Ottawa Hospital, Riverside Campus 1967 Riverside Drive, Ottawa, ON Canada K1H 7W9

**Keywords:** Magnesium, Peritoneal dialysis, Vascular calcification

## Abstract

**Background:**

Coronary artery calcification (CAC) is highly prevalent among dialysis patients and is associated with increased cardiovascular and all cause mortality. Magnesium (Mg) inhibits vascular calcification in animal and in-vitro studies but whether the same effect occurs in humans is uncertain.

**Methods:**

A single centre cross-sectional study of 80 prevalent peritoneal dialysis (PD) patients; on PD only for a minimum of 3 months. A radiologist blinded to patient status calculated their abdominal aortic calcification (AAC) scores on lateral lumbar spine radiographs, a validated surrogate for CAC.

**Results:**

Eighty patients provided informed consent and underwent lumbar spine radiography. The mean serum Mg was 0.8 mmol/L (standard deviation 0.2) and mean AAC score 8.9 (minimum 0, maximum 24). A higher serum Mg level was associated with a lower AAC score (*R* ^2^ = 0.06, unstandardized coefficient [B] = −7.81, *p* = 0.03), and remained after adjustment for age, serum phosphate, serum parathyroid hormone, low-density lipoprotein cholesterol, smoking history, and diabetes (model adjusted *R* ^2^ = 0.36, serum Mg and AAC score B = −11.44, *p* = 0.00). This translates to a 0.1 mmol/L increase in serum Mg being independently associated with a 1.1-point decrease in AAC score.

**Conclusions:**

Our findings suggest that Mg may inhibit vascular calcification. If this association is replicated across larger studies with serial Mg and vascular calcification measurements, interventions that increase serum Mg and their effect on vascular calcification warrant further investigation in the PD population.

**Electronic supplementary material:**

The online version of this article (doi:10.1186/s12882-017-0549-y) contains supplementary material, which is available to authorized users.

## Background

The mortality rate of patients on dialysis is in excess of 20% per year, with approximately one half of deaths attributable to cardiovascular disease [1, 2]. Dialysis patients have a high prevalence of traditional cardiac risk factors and experience further risk due to abnormal mineral metabolism [1]. Studies in prevalent hemodialysis (HD) patients have found that 90% of such patients have coronary artery calcification (CAC), which is associated with increased all cause and cardiovascular mortality [2–4]. Hypercalcemia, hyperphosphatemia, and hyperparathyroidism have received the most attention; several studies have demonstrated their association with accelerated vascular calcification [5]. However, there has been comparatively little exploration of the association of serum magnesium (Mg) with vascular calcification. Both in-vitro and animal studies have found that the addition of Mg to vascular smooth muscle cells inhibits the increase in mineralization associated with an osteoblastic phenotype, increases the expression of anti-calcification proteins, and down regulates pathways necessary for the development of vascular calcification [6–12]. The transient receptor potential melastin (TRPM)7 cation channel as well as the Wnt/β-catenin pathway are proposed as being essential to Mg regulating vascular calcification [7, 11]. Small observational studies and pilot studies administering Mg containing phosphate binders in dialysis populations have found a significant association between lower serum Mg levels and the progression of CAC, peripheral arterial calcification, mitral annular calcification, and atherosclerosis of the common carotid artery [13–19]. A recent study demonstrated that a higher serum Mg significantly decreased the mortality risk associated with hyperphosphatemia in HD patients [20] As well, a lower serum Mg level has been found to be associated with increased mortality in both HD and PD patients [21–27]. These cumulative results suggest a possible association between hypomagnesemia and CAC. The majority of studies have used poorly validated surrogates for CAC, [3, 28] and only one study included PD patients, [16] who are at highest risk for hypomagnesemia due to the low Mg concentration of commonly used PD solutions [29, 30]. To better characterize the relationship between serum Mg and CAC in PD patients, we performed a cross sectional study using the degree of abdominal aortic calcification (AAC) seen on a lateral lumbar spine radiograph, a validated and inexpensive surrogate for CAC [28, 31].

## Methods

### Dialysis prescription

Patients in the Ottawa Hospital Home Dialysis Program in Ottawa, Ontario, Canada were recruited from 2012 to 2014. These individuals are assessed routinely in a multi-disciplinary clinic every six weeks and recruitment was performed at a regular clinic visit. Included patients had to be on PD for a minimum of three months and had to be capable of providing informed consent. Patients on hybrid therapy (combined HD and PD) were excluded. Because the study was of cross-sectional design, all variables were measured once upon patient enrollment. Our Home Dialysis Program exclusively used PD solutions provided by Baxter Healthcare Corporation (Deerfield, Illinois) during the study period. Solutions used included Dianeal, Extraneal, and rarely Physioneal. Information on these solutions and their composition is available at: http://www.baxter.com/healthcare_professionals/products/index.html#Renal. Patients are offered a choice between continuous ambulatory peritoneal dialysis and continuous cyclic peritoneal dialysis. The dialysis programs are adjusted to deliver a minimum weekly Kt/V urea of 1.7 as per Canadian Society of Nephrology guidelines [32].

### Biochemical assays

At the time of enrolment, a patient’s serum calcium (Ca), phosphate (PO4), parathyroid hormone (iPTH), albumin, Mg and a non-fasting cholesterol profile were measured. All samples were analyzed in the Hospital Laboratory in accordance with the Hospital Laboratory Guidelines. Serum Ca and PO4 were analyzed with the Siemens Vista 1500 analyzer (Munich, Germany) (coefficient of variation (CV) for Ca 2.63% at 1.42 mmol/L and 2.1% at 2.5 mmol/L; CV for PO4 3.4% at 0.6 mmol/L and 2.4% at 1.3 mmol/L). iPTH was analyzed using the Beckman Coulter Immunoassay (Brea, California) (CV 6.9% at 2.6 pmol/L, 6.9% at 19.9 pmol/L and 5.8% at 59.3 pmol/L). Serum albumin was analyzed with the Dimension Vista (Siemens, Munich, Germany) system using an adaptation of the bromocresol purple dye binding method (CV 2.5% at 3.2 g/dL). Serum Mg was analyzed with the Dimension Vista system using a modification of the methylthymol blue complexometric procedure (CV 3% at 0.78 mmol/L and 2% at 1.93 mmol/L). Serum total cholesterol, triglycerides and high-density lipoprotein cholesterol (HDL) were measured using the Dimension Vista system (CHOL, TRIG and HDLC methods respectively). Low-density lipoprotein cholesterol (LDL) was calculated using Friedewald’s formula. Residual renal function (RRF) was calculated from a 24-h urine collection. Urine creatinine and urine urea were measured; the average of the creatinine clearance and urea clearance was taken to calculate the estimated glomerular filtration rate (eGFR) in mL/min.

### Vascular calcification

The AAC score was calculated using a lateral lumbar spine radiograph as described by Kauppila et al. from their assessment of 617 Framingham heart study participants [31]. The extent of the calcification of the anterior and posterior aortic wall was graded at each vertebral level from L1 to L4 on a 0–3 scale, yielding three different composite scores. Of these, the antero-posterior severity score, which ranges from 0 to 24 and has the highest inter-rater correlation (intra-class correlation (ICC) 0.93–0.96), was used in our study [31]. This method of determining abdominal aortic calcification has been found to be a valid surrogate for assessing CAC (area under the curve 0.78) [28, 31]. Each patient had one radiograph performed shortly after his or her enrolment. A radiologist who was blinded to each patient’s status interpreted all radiographs. A second independent, blinded radiologist interpreted the abdominal radiographs to confirm the findings of the first radiologist (ICC = 0.99 (95% confidence interval 0.98–0.99)). The ICC was calculated using a two-way mixed effects model with an absolute agreement definition.

### Statistical analysis

Data were summarized as the mean +/− standard deviation or median (interquartile range (IQR)). Univariate linear regression was performed to determine the association of serum Mg and pre-specified variables of interest with the AAC score. Based on known risk factors for vascular calcification, the following variables were examined in univariate analysis: age (per year), sex, iPTH, serum Ca, serum PO4, calculated LDL cholesterol, total time on dialysis (years), smoking history (ex or current smoker vs non-smoker), diabetes, serum albumin, and RRF. All variables with a *p*-value ≤0.2 on univariate regression were adjusted for in the multiple linear regression model. We also performed a multiple linear regression analysis where all pre-specified variables were maintained in the model. Five patients had missing LDL cholesterol values (*n* = 1, no test was performed; *n* = 4, triglycerides were too high to calculate LDL). Multiple imputation (SPSS automatic imputation method) was used to impute missing values for LDL in the multiple linear regression analysis. SPSS automatically chooses an imputation method that is most appropriate for the data and uses linear regression to impute missing continuous variables. Five imputed datasets were created and the estimates from each dataset were pooled.

We performed a sensitivity analysis where patients with an aortic calcification score of 0 were removed from the analysis. Patients with very little or no vascular calcification do not tend to develop vascular calcification over time and seem to represent a different subgroup from the general end stage renal disease population [33, 34]. For this reason, we performed a separate analysis to determine the impact upon the overall results. All analyses were performed using SPSS Statistics version 24. The reporting of this study follows the STROBE guidelines for observational studies [35].

## Results

### Baseline characteristics

**Fig. 1:**
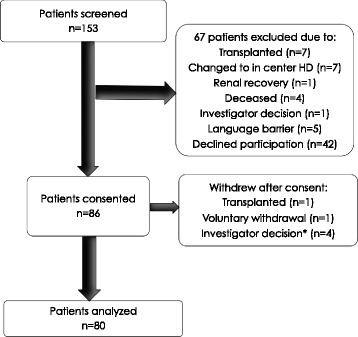
Patient selection. *These patients consented to participate but then failed to complete the required lumbar x-ray despite multiple reminders

Eighty six patients provided informed consent; 80 patients completed a lateral lumbar X-ray and were included in the primary analysis. Patient selection is outlined in Fig. 1. The baseline characteristics of included patients are outlined in Table 1. The mean age of patients was 62.8 years, 56 patients (70%) were male, 34% were diabetic and 33% were lifelong non-smokers. Mean serum Mg was 0.84 mmol/L (normal range 0.74 to 1.03 mmol/L) and mean AAC score 8.9 (minimum score 0, maximum score 24). The mean serum PO4 level was 1.70 mmol/L and mean LDL cholesterol 2.02 mmol/L. The median time on dialysis was 1.3 years (IQR 0.6 to 3.0). Twenty six patients (32.5%) had a low serum Mg (defined by <0.74 mmol/L), 42 patients (52.5%) had a normal serum Mg and 12 patients (15.0%) had a high serum Mg (defined as >1.03 mmol/L). Most patients (77.5%) were on ambulatory peritoneal dialysis (APD). Included patients used the following PD fluid combinations: 1. Dianeal only in 19 (23.8%) patients, 2. Extraneal only in 4 (5.0%) patients, 3. Dianeal and Extraneal in 54 (67.5%) patients, 4. Physioneal and Extraneal in 3 (3.8%) patients.

**Table 1:** Baseline characteristics

Total *N* = 80	
Age	62.8 (12.8)
Sex (male)	56 (70.0)
BMI (kg/metre squared)	28.33 (5.6)^a^
iPTH (pmol/L)	34.0 (28.6)
Serum Calcium (mmol/L)	2.3 (0.2)
Serum Phosphate (mmol/L)	1.7 (0.4)
LDL cholesterol (mmol/L)	2.0 (0.8)^b^
Time on dialysis (years)	1.3 (0.6, 3.0)^c^
Lifelong non-smoker	26 (32.5)
Diabetes	27 (33.8)
Serum Mg (mmol/L)	0.84 (0.2)
Number of patients with low serum Mg	26 (32.5)
Serum Albumin (g/L)	32.4 (4.5)
nPCR (g/kg/day)	0.8 (0.2)
RRF (mL/min)	4.7 (3.8)
Number of patients on a cholesterol lowering medication (statin or ezetemibe)	55 (68.8)
Number of patients on APD	62 (77.5)
Aortic calcification score	8.9 (6.9)

Continuous measurements presented as mean (standard deviation (SD)), other variables presented as N (%) unless otherwise specified

*BMI* body mass index, *iPTH* parathyroid hormone, *LDL* low density lipoprotein, *Mg* magnesium, *nPCR* normalized catabolic rate, *RRF* Residual renal function, *APD* automated peritoneal dialysis (the remainder of patients were on continuous ambulatory peritoneal dialysis)

^a^Mean value for 79 patients (1 patient had a missing value)

^b^Mean value for 75 patients (5 patients had values that were missing or could not be calculated)

^c^Median, interquartile range (IQR)

### Univariate regression

On univariate regression, age, serum phosphate, serum iPTH, LDL cholesterol, smoking history, diabetes, and serum Mg had a *p* value ≤0.2. Older age, a higher serum PO4, a lower serum Mg and a history of diabetes were significantly associated with an increased AAC score (*p* < 0.05). Higher LDL cholesterol was associated with a decreased AAC score (*p* = 0.03) (Table 2).

**Table 2:** Univariate linear regression

Variable	*R* ^2^	Unstandardized coefficient (B)	95% confidence interval (CI)	*P* value
Age	0.17	0.22	0.11, 0.33	0.00
Sex	0.005	−1.04	−4.41, 2.33	0.54
iPTH	0.02	0.03	−0.02, 0.09	0.23
Calcium	0.00	0.04	−8.55, 8.63	0.99
Phosphate	0.07	4.52	0.91, 8.13	0.02
LDL^a^	0.07	−2.19	−4.09, −0.29	0.03
Time on dialysis	0.02	−0.40	−1.10, 0.30	0.26
Smoking history	0.03	2.53	−0.73, 5.79	0.13
Diabetes	0.06	3.51	0.33, 6.68	0.03
Albumin	0.004	−0.10	−0.45, 0.24	0.56
RRF	0.001	−0.05	−0.46, 0.37	0.83
Magnesium	0.06	−7.81	−15.01, −0.61	0.03

Smoking history (ex or current vs non-smoker)

*iPTH* intact parathyroid hormone

*LDL* low-density lipoprotein

*RRF* Residual renal function

^a^75 patients included in the analysis for LDL (5 patients had values that were missing or could not be calculated). 80 patients included for all other variables

### Multiple linear regression

After adjustment for age, serum phosphate, serum iPTH, LDL cholesterol, smoking history, and diabetes, a lower serum Mg level was independently associated with a higher AAC score (Unstandardized coefficient [B] = −10.98, *p* = 0.000). This statistic translates to a 0.1 mmol/L increase in serum Mg being associated with a 1.1-point decrease in AAC score. When all pre-specified variables (Table 2) were maintained in the model regardless of the univariate analysis *p* value, the results were similar (B for Mg = −11.50, *p* = 0.000). When patients with an AAC score of 0 were excluded, *n* = 68 patients included in the analysis, the results were similar (B for Mg = −12.27, *p* = 0.001) (Table 3). Testing assumptions of the linear regression model can be found in Additional file 1.

**Table 3:** Multiple linear regression: the independent association of serum Mg with vascular calcification

Model	Adjusted *R* ^2^ of the model	Unstandardized coefficient (B) for Mg	95% CI	*P* value
1	0.34	−10.98	−17.40, −4.56	0.000
2	0.35	−11.50	−17.93, −5.06	0.000
3	0.18	−12.27	−19.54, −5.00	0.001

Model 1: Adjusted for age, serum phosphate, LDL cholesterol, iPTH, smoking history, and diabetes

Model 2: All pre-specified variables in Table 2 were included in the model

Model 3: Adjusted for the variables in model 1; patients with an aortic calcification score of 0 (*N* = 12) excluded from the analysis

*Abbreviations: LDL* low density lipoprotein cholesterol, *iPTH* intact parathyroid hormone

## Discussion

Our data demonstrate that in PD patients, a lower serum Mg is independently associated with an increased AAC score. We found that a 0.1 mmol/L increase in serum Mg is associated with a 1.1-point decrease in AAC score. This suggests that Mg may act as a possible inhibitor of vascular calcification.

Our study results are consistent with previously published observational and pilot studies in the dialysis and chronic kidney disease populations [9, 13–19]. A recent cohort study with a maximum follow up of 10.8 years found that a lower serum Mg was associated with increased mortality in PD patients [23]. Several other studies have demonstrated an association between lower serum Mg and mortality among dialysis patients [21, 22, 24–27] This association could potentially be attributable to low serum Mg causing accelerated vascular calcification. Increasing data from in-vitro and animal studies support the assertion that Mg acts as an inhibitor of vascular calcification [6–12]. The addition of Mg to vascular smooth muscle cells increases the expression of anti-calcification proteins, such as matrix G1a, bone morphogenetic protein-7 and osteopontin, and inhibits an osteoblastic transformation [7, 11]. As well, the addition of Mg to vascular smooth muscle cells down-regulates the Wnt/β-catenin pathway. This pathway is essential for the osteogenic transformation of pluripotent mesenchymal cells and is activated during the development of vascular calcification [11]. The mechanism by which Mg regulates vascular calcification may involve the transient receptor potential melastin (TRPM)7 cation channel as inhibition of TRPM7 negates the anti-calcification effects of Mg [7, 11]. Mg may also inhibit vascular calcification by suppressing PTH, which has been found in animal models to increase vascular calcification [36, 37]. Among PD patients, an inverse correlation between PTH and serum Mg, independent of Ca concentration, has been demonstrated in several studies [38–41]. However, in our cohort of PD patients, we did not find a significant, independent association between serum Mg and PTH (data not shown). To our knowledge, our study is the largest thus far in the PD population (previously published PD study *n* = 44; outcome of peripheral arterial calcification [16]), and the largest study with a vascular calcification outcome that is a validated surrogate for CAC.

AAC is a valid surrogate marker as it correlates with CAC and is associated with increased all-cause and cardiovascular mortality [28, 42, 43]. Among diabetic patients, reported hazard ratios for all-cause and cardiovascular mortality were 1.7 and 1.9 respectively when the AAC score was examined as a continuous variable [42]. Cardiovascular mortality has also been found to increase in a graded fashion with increasing tertile of AAC score [43].

Our study population had a mean serum Mg of 0.84 mmol/L, and over 32% of our cohort had low serum Mg (defined as <0.74 mmol/L). The mean serum Mg was similar in a study by Fein et al. (0.8 mmol/L) examining the association of serum Mg with mortality in PD patients [23]. Comparatively, studies examining serum Mg in an HD population found a higher mean Mg level of 1.14 mmol/L [24], 0.92 mmol/L [26], and 0.86 mmol/L [21]. The high prevalence of hypomagnesemia in our cohort of PD patients could be due to the continuous nature of PD, coupled with the low magnesium concentration of commonly used PD solutions (Mg concentration ranging from 0.25 to 0.75 mmol/L). Patients on automated PD overnight using a PD solution containing 0.25 mmol/L of Mg along with a day time dwell of an icodextrin solution, (the most commonly used regimen at our institution), have an overall transperitoneal Mg loss of 3.26 mmol per 24 h [29]. Magnesium losses in the dialysate are compounded by the significant restrictions of a renal diet. Taken together, this highlights the unique risk of hypomagnesermia in PD patients, making them an ideal population for testing interventions targeted at increasing serum Mg and examining the effect on vascular calcification.

Our study has some important limitations. Due to the cross-sectional nature of the study, we can only determine association and not causation. Residual confounding is possible. However, we were able to adjust for important confounders associated with vascular calcification, such as serum PO4 and age [3], and our findings were consistent across unadjusted, adjusted and sensitivity analyses. A low serum Mg may be a marker of generalized malnutrition [44, 45] and inflammation, which are both associated with increased vascular calcification [46, 47]. We did not directly measure any inflammatory markers; however, albumin is a recognized surrogate for inflammation and malnutrition. On univariate analysis, albumin was not associated with the AAC score, and the results of the multivariate analysis were not attenuated upon adjustment for albumin. We only measured serum Mg at one time point; it is possible that a single measurement may not be reflective of an individual’s overall Mg status. The imaging technique used in our study did not allow us to reliably differentiate between medial (AIM) and intimal calcification (AIC). Both types of vascular calcification occur commonly in dialysis patients, often co-existing in the same patient, and are associated with increased mortality. However, among dialysis patients, AIC has been found to be associated with worse survival when compared to AIM. As well, the clinical consequences of AIC and AIM differ. While AIC represents advanced atherosclerosis that is associated with the development of plaques and occlusive disease, AIM causes arterial stiffness, increased pulse pressure and left ventricular hypertrophy [4, 48, 49].

## Conclusions

In conclusion, our results support the assertion that Mg may inhibit vascular calcification, a condition that is highly prevalent in the dialysis population and is associated with increased mortality [2–4]. If our results can be duplicated in large observational studies with repeated serum Mg and vascular calcification measurements, interventions, such as Mg supplementation in hypomagnesemic patients, the use of Mg based phosphate binders, or the use of PD solutions with a higher Mg concentration and their effect on vascular calcification warrant testing in the PD population. Such interventions would be easy to administer and would carry minimal side effects.

## Additional file

Additional file 1: Testing assumptions of the linear regression model. (DOCX 101 kb)

## Abbreviations

AAC: Abdominal aortic calcification
APD: Ambulatory peritoneal dialysis
Ca: Calcium
CAC: Coronary artery calcification
HD: Hemodialysis
ICC: Intra-class correlation
LDL: Low density lipoprotein
Mg: Magnesium
PD: Peritoneal dialysis
PO4: Phosphate
TRPM7: The transient receptor potential melastin
