# Supplementary material for: Lower serum magnesium is associated with vascular calcification in peritoneal dialysis patients: a cross sectional study
Source: BMC Nephrol. 2017 Apr 6;18:129. doi: 10.1186/s12882-017-0549-y (PMC5382660; doi:10.1186/s12882-017-0549-y)
Supplement: Additional file 1: — Testing assumptions of the linear regression model. (DOCX 101 kb) [file 12882_2017_549_MOESM1_ESM.docx]

Additional file 1: Testing Assumptions of the Linear Regression Model


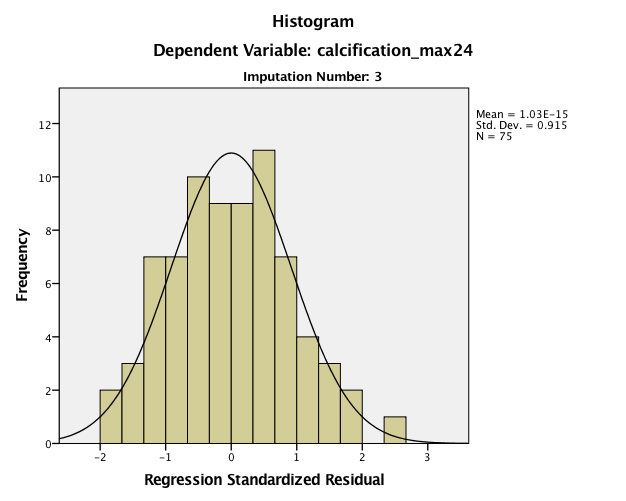


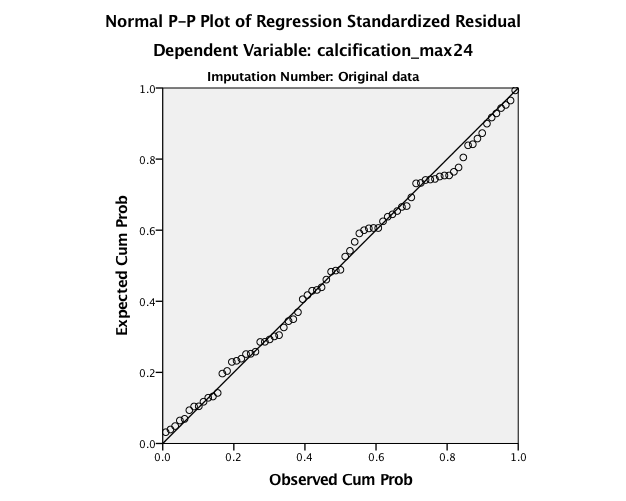


Durbin Watson test: 2.08

Collinearity Tolerance statistics all <0.9.

Residuals Statistics:

Maximum Cook’s Distance 0.161 (<1)
